# A persistent influence of supernovae on biodiversity over the Phanerozoic

**DOI:** 10.1002/ece3.9898

**Published:** 2023-03-16

**Authors:** Henrik Svensmark

**Affiliations:** ^1^ National Space Institute Technical University of Denmark Lyngby Denmark

**Keywords:** biodiversity, evolution, macrodiversity, marine‐genera, Phanerozoic, supernova

## Abstract

It is an open question what has constrained macroevolutionary changes in marine animal diversity on the time scale of the Phanerozoic. Here, we will show that supernovae appear to have significantly influenced the biodiversity of life. After normalizing diversity curves of major animal marine genera by the changes in the area of shallow marine margins, a close correlation between supernovae frequency and biodiversity is obtained. The interpretation is that supernovae influence Earth's climate, which controls the ocean and atmospheric circulation of nutrients. With this, supernovae influence ocean bioproductivity and are speculated to affect genera‐level diversity. The implication is a surprisingly influential role of stellar processes on evolution.

## Graphical abstract

It shows the correlation between diversity and supernovae frequency.

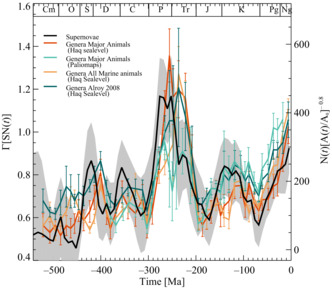

## 1 INTRODUCTION

For nearly four billion years, life evolved from single cells to multicellular life whose diversity gets documented in the fossil record and present‐day richness of life. Sepkoski comprised the first compendium of marine animal families and genera over the Phanerozoic (Sepkoski et al., 1981), from which he could demonstrate temporal changes in biodiversity. A fundamental question is why biodiversity changes, particularly what processes shape biodiversity patterns. Decades of research have gone into understanding changes in biodiversity over geological time (Alroy, 2010a, 2010b; Alroy et al., 2008; Bambach, 1999; Bambach et al., 2002; Benton, 1995; Foote, 2000; Sepkoski, 1984; Sepkoski et al., 1981; Stanley, 2008; Valentine, 1969). One idea is that processes are operating at the population level (microevolution and population ecology) and are thereby responsible for long‐term and large‐scale macroevolutionary and macroecological changes. Another nonexclusive possibility is mechanisms operating at the ecosystem level may explain these phenomena. This contribution will highlight two possible causes of changes in biodiversity: shallow marine areas and supernova (SN) frequency.

Most marine life resides on shallow marine margins, and large changes in sea level over the Phanerozoic may result in flooding of the continents. This flooding opens up new isolated areas where species can evolve (Flessa & Sepkoski, 1978; Peters, 2008; Peters & Foote, 2001; Schopf, 1974; Simberloff, 1974). However, the relevance of supernovae frequency for biodiversity may be unexpected, but the idea is that they affect climate. From an astrophysical point of view, supernovae constitute a major source of chemical elements in our Galaxy and deliver energy and momentum to the interstellar medium, each with an energy release of the order of 10^44^ Joules. Fortunately, the probability of an SN exploding very close to the Solar system (~10 pc) is tiny. A close SN would have devastating effects on the biosphere. It is estimated to occur much less than once per 100 Myr (Clark et al., 1977), and so far, no geological evidence exists of a very close SN encounter (Fields et al., 2019). Therefore, the explosive energy and direct radiation from a single SN will not generally provide a continuous effect on the biosphere. Here, the relevant supernovae are core‐collapse supernovae (SNe II, SNe Ib, and SNe Ic). These are massive stars (>8 $M_{⊙}$) found in star‐forming regions, with a short lifetime of ~2–30 Myr. Since these stars form close to the Galactic mid‐plane, they also explode close to their birthplace. Core‐collapse supernovae are the most frequent supernovae responsible for about 85% of the SNe in the Galaxy (Tsujimoto et al., 1995). The important feature of SNe is the acceleration of protons and heavier elements by the expanding shock front of supernovae remnants, which causes the local cosmic ray spectrum. After leaving the supernova remnants, cosmic rays are transported by diffusion in the interstellar medium, with a typical lifetime of 10 Myr before they escape the Galaxy. While in the interstellar media, some cosmic rays will enter the solar system. Here, they interact with the solar wind and may end up in Earth's atmosphere, becoming the primary source of atmospheric ionization. The connection of cosmic rays to climate initially concerned changes in solar activity. The idea was that cosmic rays ionization could influence Earth's cloud cover and affect the energy budget. Although originally controversial, the link between cosmic rays and clouds has a basis in observation and extensive experiments (Kirkby et al., 2011; Svensmark et al., 2007, 2009; Svensmark & Friis‐Christensen, 1997). It is now known that atmospheric cosmic ray ionization is instrumental in forming nano‐sized aerosols, which may grow to cloud condensation sizes (~50 nm) and influence cloud properties (Kirkby et al., 2011; Svensmark et al., 2007, 2017; Svensmark & Friis‐Christensen, 1997). A high flux of cosmic rays means increased cloudiness and a colder climate. In 2002 Nir Shaviv investigated whether the mechanism could also operate on astronomical time scales by noting that over ~230 Myr, the solar system orbit around the center of our Galaxy and moves in and out of star‐forming regions in connection with the spiral arm structure of the Galaxy. During this journey, Earth encounters large changes in the intensity of the cosmic ray flux, resulting in variations in atmospheric ionization of the order 100% (Shaviv, 2002).

Of course, there are several possible climate‐forcing agents over millions of years. The most straightforward forcing is the long‐term increase in solar luminosity, which in the previous 600 Myr rose by ~5% (Bahcall et al., 2001). At the same time, greenhouse forcing from CO_2_ decreased as the concentration declined. Interestingly, the climatic cooling of the long‐term decrease in CO_2_ mostly cancels out the increasing solar luminosity (Shaviv et al., 2023).

That still leaves significant changes in Earth's climate over the Phanerozoic period (last 542 Myr), from warm greenhouse climates to icehouse climates. In accordance with the cosmic ray theory, Earth experienced cold glacial periods when the local supernova frequency was high, i.e., high cosmic rays and warm climates when the flux was low (Shaviv, 2002, 2003; Shaviv et al., 2023; Shaviv & Veizer, 2004; Svensmark, 2012). These results suggest that changes in supernovae frequency and, thereby, changes in cosmic rays have significantly influenced the Phanerozoic climate (Svensmark, 2012, see figure 18; Svensmark, 2022, see figure 3; Shaviv et al., 2023, see figure 3).

The influence of supernovas on Earth's climate appears to be so considerable that it also affects the conditions of life. For example, one finds a close correlation between supernova rates and the burial of organic matter in ocean sediments during the last 500 Ma (Svensmark, 2022). The interpretation is that supernovae rates influence climate and, thereby, the atmosphere–ocean circulation. Atmospheric and oceanic circulation is vital for providing nutrients to organisms, and nutrient concentrations control bioproductivity. Therefore, the understanding is that nutrient delivery results in a high bioproductivity, which gives a larger fraction of organic material buried, in concert with the changes in supernovae rates. In this way, supernovae rates regulate climate and the total energy available to the biological systems. As will be shown, supernovas ultimately influenced the diversity of marine life.

## 2 MATERIALS AND METHODS

### 2.1 Diversity curves

**FIGURE 1:**
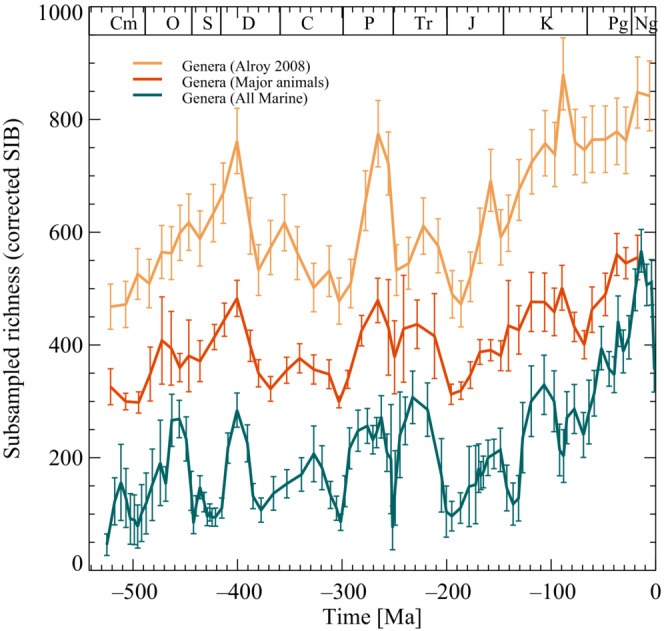
Three curves illustrate the temporal evolution of the diversity of Phanerozoic marine animal genera. The yellow top curve is from Alroy et al. (2008) (offset +200 on the *y*‐axis) and depicts the genus‐level marine invertebrate. The middle diversity curve (brown) is major marine animals (offset by +250), and finally, the bottom (green) diversity curve is all marine animals (see section 2: materials and methods). The two bottom curves were calculated using “shareholder‐quorum‐subsampling” (SQS) with q=0.5. The error bars are one‐sigma uncertainties. Abbreviations for geological periods are Cm, Cambrian; O, Ordovician; S, Silurian; D, Devonian; C, Carboniferous; P, Permian; Tr, Triassic; J, Jurassic; K, Cretaceous; Pg, Palaeogene; Ng, Neogene.

Genus diversity curves of Figure 1 use data from the “The Paleobiology Database” https://paleobiodb.org. This work used the statistical methods collected in the R‐program package “divDyn” to construct diversity curves of major animal marine genera (Kocsis et al., 2019) where programs and documents are available at https://github.com/divDyn/r\_package. The diversity curves are formed by using the statistical shareholder‐quorum‐sampling (SQS) method (Alroy et al., 2008). This method estimates diversity by random sampling fossil collections until each temporal bin contains the same number of specimens. The statistical procedure is then repeated 1000 times, from which a mean diversity curve is obtained together with the standard variation.

The diversity curve Figure 1 (red curve) of major marine animals higher uses the following taxa: Anthozoa, Bivalvia, Brachiopoda, Bryozoa, Cephalopoda, Chondrichthyes, Conodonta, Crinoidea, Echinoidea, Gastropoda, Graptolithina, Linguliformea, Ostracoda, Trilobita, and is similar to the Alroy (2010a) diversity curve. These data consist of 253,217 entries in the database. The data file and R‐program are stored in DRYAD (See data availability statement).

The diversity curve Figure 1 (green curve) of all marine animals is based on an extract from “The Paleobiology Database” on 9 April 2022 (1.5 Gbyte). These data consist of 926,581 entries in the database. The data file and R‐program are stored in DRYAD (See Data Accessibility Statement).

### 2.2 Supernovae rates

The data used in Figure 3 are supernovae frequencies based on three data sets of open stellar clusters in the solar neighborhood. A description of the methods used on the open stellar clusters data to obtain the supernovae frequencies is available in Svensmark (2012) and the numerical values of supernovae frequencies, used in Figure 3, are in the supplementary information of Svensmark (2022).

### 2.3 Area of shallow marine margins

The fractional area of shallow marine margins was extracted from 24 paleogeographical maps covering the last 400 Ma (Cao et al., 2017). Data for the global, northern, and southern hemispheres and tropics are stored in DRYAD (See data availability statement).

## 3 RESULTS

Due to uneven sampling bias and incomplete rock and fossil records, a universally accepted genus diversity curve does not exist. Consequently, diversity curves can vary significantly due to uneven sampling and preservation. Nonetheless, there are common identifiable long‐time features of marine animal genus diversity—an initial (uneven) rise to a Paleozoic plateau followed by a drop in the Early Mesozoic and a final increase towards a maximum of the present. Alroy et al. (2008) have addressed shortcomings in fossil records by proposing statistical methods giving more robust diversity curves. This work used the statistical methods collected in the R‐program package “divDyn” to construct diversity curves of major animal marine genera (Kocsis et al., 2019). Figure 1 shows three “shareholder‐quorum‐subsampling” (SQS) diversity curves. The yellow top curve is data from Alroy et al. (2008) of marine invertebrate groups (offset by +200). The middle brown curve shows major marine animals (offset by +250). Finally, the bottom diversity curve is all marine animals extracted from “The Palio‐Database” (further information, see section 2: materials and methods). Note that although there are differences, there are overall agreements. The difference between the yellow diversity curve in Figure 1 and the classical Sepkoski curve (Sepkoski et al., 1981) is the removal of biases with standardized sampling and sampled‐in‐bin counting of occurrence data (Alroy et al., 2008). Additional dissimilarities between the curves are differences in taxa and the number of entries in the database at the time of extraction. As will be shown, these differences will not seriously affect the results obtained here. Diversity is believed to be affected by a number of external causes. For example, climate change, sea level, anoxic events, major volcanic eruptions, large impacts, and plate tectonics are all suggested to influence the evolution of life on diverse timescales (Cañón‐Tapia & Walker, 2004; Hallam & Wignall, 1997; House, 2002; Keller, 2005; Peters, 2008; Racki, 2005; Rich et al., 1986; Smith & Pickering, 2003; Walliser, 1996). This paper will focus on the long‐term changes in diversity induced by variations in sea level and supernovae frequency. With this focus, it is not the aim to exclude other effects mentioned above on evolution. Instead, by building upon empirical relations, the justification is the level of correlations or consistency obtained by concentrating on the role of sea level and supernovae frequency on diversity.

**FIGURE 2:**
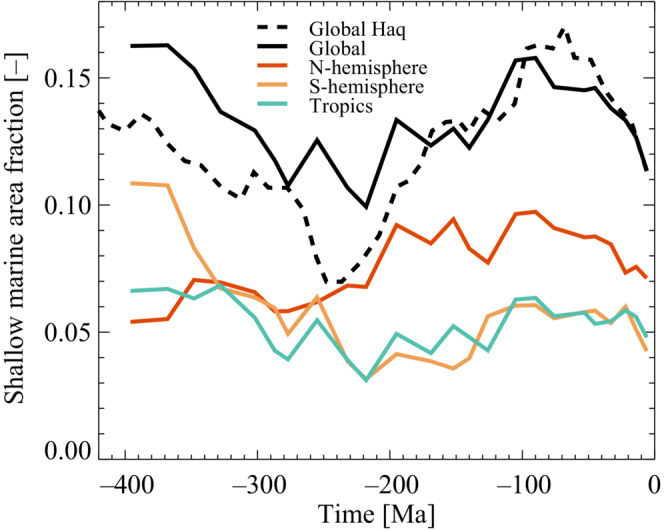
Change in the fractional shallow marine area as a function of time during the last 400 Ma. The black dashed curve is based on the Exxon on‐lap reconstruction of sea level (Haq et al., 1987; Haq & Schutter, 2008; see text). Paleogeographic maps (Cao et al., 2017) give the global fractional shallow marine area (black solid curve), the fractional shallow marine area of the northern (southern) hemisphere, and brown (yellow) curves, and finally, the fractional shallow marine area of the tropical region (green curve).

A well‐founded ecological pattern is that more species are found when sampling a larger area (Arrhenius, 1921; Connor & McCoy, 1979; Gleason, 1922; May & Stumpf, 2000; Ricklefs & Lovette, 1999; Rosenzweig, 1995; Williams, 1943). The empirical relation between species and area is,

(1) $$N\propto A^{\alpha },$$

where N is the number of species/genera, and A denotes the area. α is an exponent closer to 1.0 than to 0 for larger areas, i.e., inter‐provinces, α≈0.8 (Rosenzweig, 1995). Most marine life lives in shallow marine margins along the continental shelf, and variations of this area are important for the presiding marine diversity of the major animal groups. The above species–area relations, Equation (1), are normally used to count species at a particular point in time, but in this study, the shallow marine area will change over the Phanerozoic time scale. Therefore, the task is to reconstruct temporal changes in shallow marine areas. This reconstruction is possible by knowing the variations in sea level responsible for flooding the continental margins and shelves. Based on the average topography and bathymetry, a relationship between sea level and flooding area is obtained by the linearization of the global hypsometric curve around the present‐day sea level, written as

(2) $$A\left(t\right)=\mathrm{k\sigma}\left(t\right),$$

where $A\left(t\right)$ is the temporal change in shallow marine area, and $k=2.02\cdot 10^{8}$ km^2^/km is the hypsometric gradient, and $\sigma \left(t\right)$ is the temporal change in sea level (van der Meer et al., 2017). Sea‐level changes are estimated using seismic stratigraphy from Haq et al. (1987) and Haq & Schutter (2008). The cause of changes in sea level on time scales longer than ca. 1 Myr is related to changes in ocean basin volume caused by slow variations in sea‐floor spreading rates or ocean ridge lengths and the size of ice sheets (Miller et al., 2005). Combining the sea‐level data with Equation (2) gives an estimate of global relative changes in shallow marine areas. Figure 2 shows the fractional change in the shallow marine area based on Equation (2) and the sea‐level data from Haq & Schutter (2008) and shown as the black dashed line. It is also possible to obtain spatial information on shallow marine areas by using results from global paleogeographic maps and paleo‐environmental data for the last 402 million years (Cao et al., 2017). Based on 24 maps covering the period from 396 to 6 Ma, it is possible to extract the temporal variation in shallow marine areas. Figure 2 shows the temporal evolution of the relative shallow marine area for four regions of the Earth over the last 400 Ma. (1) Global, black solid curve, (2) northern hemisphere, brown curve, (3) southern hemisphere, yellow curve, and (4) tropics, green curve. Notice that the two global reconstructions, solid and dashed back curves agree reasonably well. Also, note that 400 Ma years ago, most of the continents were in the southern hemisphere. Therefore, this hemisphere had the largest shallow marine areas, in contrast to the present time, where most continents are in the northern hemisphere.

If diversity over the Phanerozoic eon is denoted $N\left(t\right)$ and expressed as a function of changes in supernova rates and shallow marine area, $A\left(t\right)$, it leads to

(3) $$N\left(t\right)=\Gamma \left(\mathrm{SN}\left(t\right)\right){\left[\frac{A\left(t\right)}{A_{0}}\right]}^{\alpha }+\epsilon \left(t\right)$$

**FIGURE 3:**
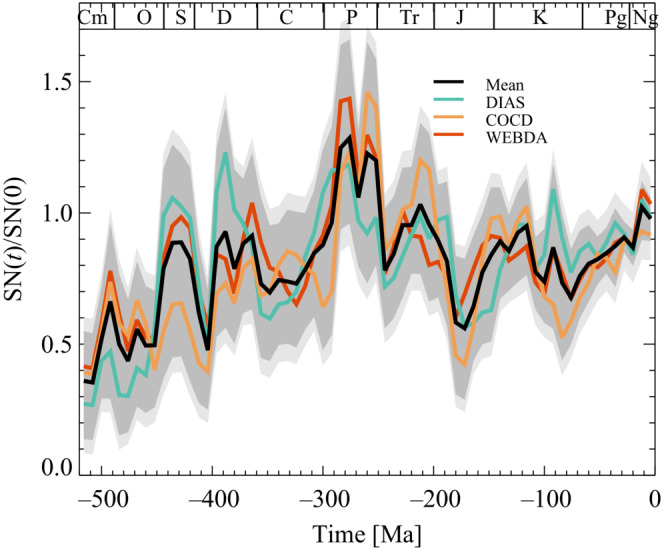
Variation in relative supernova frequency using three open cluster catalogues. (1) WEBDA catalogue (273 clusters with distance from solar system ≤850 pc and age ≤ 520 Myr). The DIAS (Dias et al., 2010) catalogue (224 clusters with a distance of 850 pc and age ≤ 520 Myr), and finally, the Kharchenko et al. catalogue (Kharchenko et al., 2005; 258 clusters with a distance ≤850 pc and age ≤ 520 Myr). The black curve is based on the average of the three catalogues. The gray band is one σ uncertainty, random normal distribution (gray band), or a random Poisson distribution (light gray band; Svensmark, 2012; Provide details on the uncertainties).

$\Gamma \left(\mathrm{SN}\left(t\right)\right)$ is a function that comprises the effect of the supernova rate on diversity. $\epsilon \left(t\right)$ is a noise term that includes the data's uncertainty and the effect of the approximation, e.g., other features affecting diversity besides supernovae and shallow marine areas. Figure 3 displays the change in supernova frequency over the last 500 Ma. Here, the supernova frequency is reconstructed by estimating star formation in the solar neighborhood based on open stellar clusters (Svensmark, 2012). As can be seen from Figure 3, the supernova rate is quite variable, and a sudden change in the supernova rate may induce a response in diversity over a time scale λ. Studies of origination and extinction dynamics in the marine fossil record suggest that the rebound time scale is 10–40 Myr, where the 40 Myr long timescale is for large events of Permian–Triassic mass‐extinction size (Alroy, 2008). Including this time scale and using the fact that the diversity $N\left(t\right)$ can only depend on past changes of $\mathrm{SN}\left(t\right)$ leads to the simple function

(4) $$\Gamma \left(\mathrm{SN}\left(t\right)\right)=c\int_{-\infty }^{t}\mathrm{SN}\left(t^{\prime }\right)\exp\left[-\frac{\left(t-t^{\prime }\right)}{\lambda }\right]{\mathrm{dt}}^{\prime },$$

where c is a constant and $\lambda \in \left[10,40\right]$ Myr. The area function ${\left(A\left(t\right)/A_{0}\right)}^{\alpha }$ is the normalized shallow marine area.

The area function can be expressed as a function of sea level using Equation (2) as

(5) $${\left[\frac{A\left(t\right)}{A_{0}}\right]}^{\alpha }={\left(1+\left[\frac{\sigma \left(t\right)-\sigma_{0}}{\sigma_{0}}\right]\right)}^{\alpha },$$

where $\sigma_{0}$ is the sea level at t=0 (present time). Miller et al. (2005) estimated that the amplitudes of the Haq et al. (1987) and Haq & Schutter (2008) sea‐level curve was approximately a factor of 2 to large and is therefore scaled down by this factor.

By dividing genera by the area function, the effect of astrophysics on genera can be isolated and assume the form,

(6) $$\Gamma \left(\mathrm{SN}\left(t\right)\right)=N\left(t\right){\left[\frac{A\left(t\right)}{A_{0}}\right]}^{-\alpha }-\;\epsilon^{\prime }\left(t\right),$$

and where $\epsilon^{\prime }\left(t\right)$ again is a noise term.

Combining Equations (4) and (6), one gets

(7) $$\;N\left(t\right){\left[\frac{A\left(t\right)}{A_{0}}\right]}^{-\alpha }=c\int_{-\infty }^{t}\mathrm{SN}\left(t^{\prime }\right)\exp\left[-\frac{\left(t-t^{\prime }\right)}{\lambda }\right]{\mathrm{dt}}^{\prime }+\;\epsilon^{\prime }\left(t\right).$$

The left side depends only on terrestrial quantities, and the right side depends only on astrophysical quantities.

**FIGURE 4:**
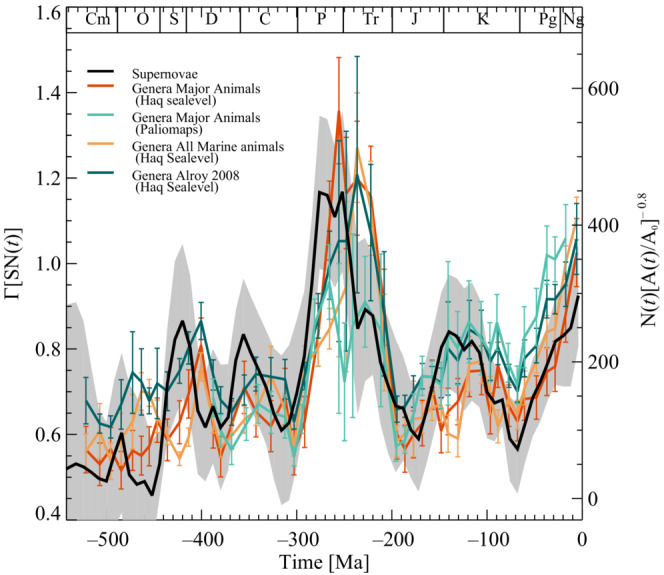
Variations in relative supernova history compared with genera of major marine animal groups. The black curve is based on the supernova rates and is given by Equation (4). The brown and light green curves show major marine animal genera normalized with the area of shallow marine margins based on Haq et al. (1987), Haq & Schutter (2008) and Cao et al. (2017), respectively. The dark green curve is based on the marine invertebrate genera‐level diversity curve of Alroy et al. (2008). Finally, the dark green curve is all marine animals normalized with the area of shallow marine margins based on Haq et al. (1987) and Haq & Schutter (2008). The exponent used in Equation (6) is α=0.8. The gray area is the 1−σ variance of the supernovae calculated from a Monte Carlo simulation. The error bars on the genera curves show a minimum 1−σ uncertainty since an error estimate is unavailable for the areas.

Figure 4 shows the result of normalizing the three diversity curves of Figure 1 with the shallow marine margins according to the left‐hand side of Equation (7) and compared with the right‐hand side (astrophysics) of Equation (7) and shown as the black curve with λ=10 Myr and α=0.8. The shallow marine margins are either based on the Haq & Schutter (2008) sea‐level curve or the paleogeographic maps Cao et al. (2017). The brown curve is normalized major marine animal diversity using the Haq et al. sea‐level curve (Haq et al., 1987; Haq & Schutter, 2008). The light green curve is similar to the brown curve but uses global paleogeographic maps (Cao et al., 2017). The yellow curve is all marina animal's genera normalized with the Haq et al. sea‐level curve. Finally, the dark green curve is the marine invertebrate genera‐level diversity curve of Alroy et al. (2008), again normalized using the Haq et al. (1987) and Haq & Schutter (2008) sea‐level curve. Notice the overall agreement between the four normalized diversity curves and the black curve. Table 1 shows the correlations together with their significance intervals between the normalized diversity curves, and the function $\Gamma \left(\mathrm{SN}\left(t\right)\right)$ for various values of λ and α. The significance intervals shown are ±1σ.

**TABLE 1:** Correlation between the genera data sets shown in Figure 1 normalized with the area of the shallow marine areas obtained from either paleo maps or Haq sea level and the supernova function $\Gamma \left(\mathrm{SN}\right)$ (see Equation (7)). For a variation of the parameters α (column 2) and λ (column 3). Column 3 is the correlation coefficient with 1σ confidence intervals. Column 4 is the variance explained by the correlation.

Data set	λ [Myr]	α [−]	Correlation [−]	Variance explained [%]
Genera major animals Normalized with Haq et al. sea level	10	1.00	$0.78_{0.64}^{0.87}$	61.2
	10	0.80	$0.77_{0.62}^{0.86}$	59.3
	10	0.60	$0.72_{0.54}^{0.83}$	51.3
	20	1.00	$0.86_{0.76}^{0.92}$	73.6
	20	0.80	$0.83_{0.72}^{0.90}$	69.0
Genera major animals Normalized using Paliomaps	10	1.00	$0.51_{0.21}^{0.72}$	26.1
	10	0.80	$0.48_{0.18}^{0.70}$	23.2
	10	0.60	$0.45_{0.14}^{0.68}$	20.2
	20	1.00	$0.49_{0.19}^{0.71}$	23.9
	20	0.80	$0.46_{0.14}^{0.68}$	20.7
Genera all marine animals Normalized with Haq et al. sea level	10	1.00	$0.55_{0.32}^{0.72}$	30.7
	10	0.80	$0.52_{0.28}^{0.70}$	27.5
	10	0.60	$0.48_{0.23}^{0.67}$	23.0
	20	1.00	$0.64_{0.44}^{0.78}$	41.0
	20	0.80	$0.60_{0.38}^{0.75}$	35.8
Genera Alroy 2008 Normalized with Haq et al. sea level	10	1.00	$0.71_{0.54}^{0.83}$	51.0
	10	0.80	$0.69_{0.51}^{0.82}$	47.8
	10	0.60	$0.65_{0.45}^{0.79}$	42.1
	20	1.00	$0.78_{0.64}^{0.87}$	61.4
	20	0.80	$0.75_{0.59}^{0.85}$	55.8

**FIGURE 5:**
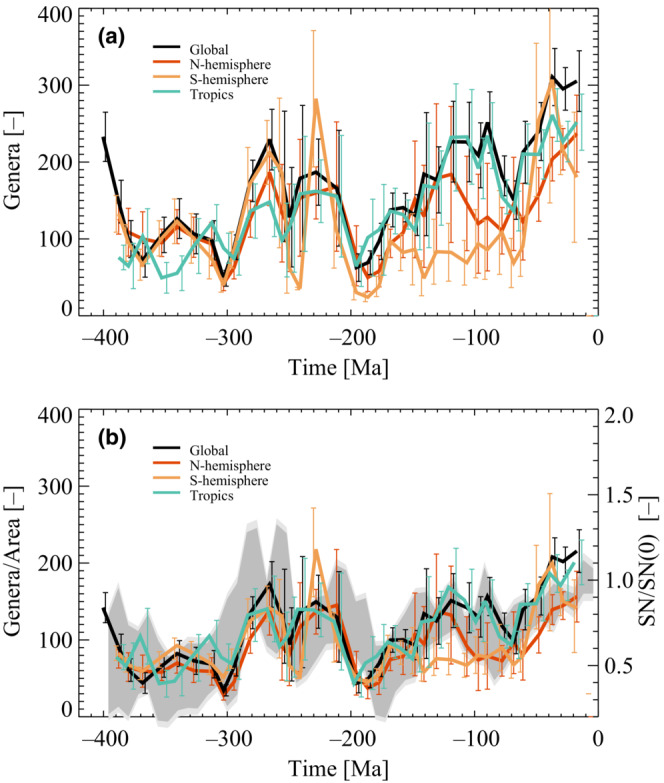
(a) Genera‐level diversity curves for major marine taxonomic groups during the last 400 Ma for different parts of the Earth. The black curve is global, the brown curve is for the northern hemisphere, the yellow curve is for the southern hemisphere, and the green curve is for the tropics. Error bars are 1−σ uncertainty. (b) Same data as in (a) but normalized with the change in areas shown in Figure 2. Notice that the curves converge towards a common variation resembling the change in supernovae. Error bars are 1−σ uncertainty. The gray band is an outline of the supernova frequency (see Figure 3).

It is possible to test further the apparent relation between diversity and supernova rates and area. The fossil records make it possible to construct genera‐level diversity curves for the northern and southern hemispheres and the tropical region (30S< latitude <30*N*), as seen in (Figure 5a). Notice the relatively large scatter between the curves, for example, diversity is increasing for the northern hemisphere in contrast to the southern hemisphere. Normalizing the diversity curves with their corresponding shallow marine areas (Figure 1) gives the result seen in (Figure 5b). It is evident that the curves are now less scattered and that the changes more closely resemble the supernova frequency (the gray band in Figure 5, where a small linear trend is added).

## 4 DISCUSSION

Unexpectedly, the present results suggest that marine animals' diversity is influenced by changes in shallow marine areas and supernova frequency. Flooding part of the continents increases the size of shallow margins, thereby creating new niches where species can develop. The present work does not determine what type of speciation occurs. The flooding of continents suggests isolations of species where no gene flow between two populations initially of the same species due to physical separation (allopatric speciation; Croizat, 1962; Howard, 2003). However, speciation can also occur even though species occupy the same geographical area and in the presence of gene flow (sympatric speciation; Bolnick & Fitzpatrick, 2007; Sousa & Hey, 2013). Nonetheless, increasing the shallow marine areas by flooding continents should raise the geographical heterogeneity of possible habitats and increase the number of species (Rosenzweig, 1995).

What is the relation between diversity and supernova‐induced climate? Climate influences temperatures, which affects ecosystems. Species must adapt or migrate if possible. So climate change transforms ecosystems, something that Darwin recognized and more recently by Vrba (Vrba, 1993) with the theory of turnover pulses. But there may be an additional effect of a changing climate.

In 2012 a connection between supernovae and life was suggested, which provided a rigorous derivation of the change in supernova frequency over the Phanerozoic (Svensmark, 2012). Since then, new results have made it possible to provide a better foundation on which the present work builds. The interpretation is that changes in cosmic rays determine climate, and climate is responsible for the available kinetic energy in the ocean–atmosphere system. Climate thereby determines the mixing and transport of fundamental nutrients like iron, nitrogen, phosphor, and carbon in the oceans and atmosphere. Support for this scenario comes from trace elements stored in pyrite formed in black shale. Here, the concentration of trace elements in pyrite is a proxy of nutrient availability in the oceans and correlates closely with cosmic ray changes over the Phanerozoic (Svensmark, 2022). Nutrients are of fundamental importance for biological systems; for example, phosphorous is a limiting factor of bioproductivity through time (Guidry & Mackenzie, 2000). Climate and, therefore, nutrient availability is a function of the flux into the oceans from the land by river runoff and windblown dust. Finally, ocean mixing brings nutrients to the surface waters along the continental shelves. The understanding is, therefore, that supernovae, through climate, drive the circulation and mixing of nutrients and are responsible for gross primary bioproductivity and, thereby, the energy flow through the biological systems. This energy is a fundamental quantity that limits the possible sizes of populations in ecosystems. Initially, speculation was that more bioproductivity would raise diversity (Rosenzweig, 1995). However, there are examples contradicting this. For instance, in the ocean, the bioproductivity decreases with depth, but there is a local maximum in diversity at intermediate depth (Haedrich et al., 1980; example with 3 major taxa represented [decapod crustacean, echinoderms, and fishes]). But, as Rosensweig notes (Rosenzweig, 1995, p. 347), it is unknown what the effect of higher bioproductivity would be on diversity over millions of years when evolution is taken into account. Perhaps the present result is an example where increased nutrients lead to higher bioproductivity and finally an increase in diversity.

The results depicted in Figure 4 are consistent with this hypothesis, i.e., that supernovae influence climate, which influences circulation and thereby the flow of nutrients, which impacts the bioproductivity and finally affects the genera‐level diversity.

Regional genera‐level diversity curves for the southern, tropics, and northern hemispheres further test the climate and area relation. Figure 5a shows the genera‐level diversity curves for the various regions, which display a relatively large scatter. Normalizing the genera‐level diversity curves with their respective areas, seen in Figure 2, one gets the results observed in (Figure 5b). In this case, the scatter between curves in 5 gets significantly smaller, and the functional forms become close to the supernova variation, as one would expect if supernova rates are part of a common cause for changes in diversity. Although the agreement is not perfect, one should remember that the statistical uncertainty in fossil data increases as the globe gets further divided. For example, one sees an apparent deviation of the southern hemisphere between 200 and 100 million years ago. However, at the same time, the continents are mainly in the northern hemisphere, perhaps resulting in poorer statistics for the southern hemisphere.

## 5 CONCLUSION

A close correlation appears between changes in supernovae frequency and changes in the diversity of marine animal genera over the Phanerozoic after normalizing the diversity with the variations in the area of shallow marine margins. This result suggests that supernova frequency and the area of shallow marine margins have been vital in shaping the diversity of marine life. The latter is not entirely unexpected since the flooding of continents opens up new regions where new species can evolve. The study builds on empirical relations with highly significant correlations. Although the correlations do not mean that no other influences are affecting diversity, they suggest an effect of supernovae on diversity. If true, it is in itself a remarkable result. But the idea of a role for supernovas in biodiversity is based on more than just the demonstrated correlations. Over the last two decades, there has been significant progress in understanding that supernovae, a source of cosmic rays, have an important influence on climate, both on short time scales and geological timescales. Adding to a consistent picture is that supernovae frequency correlates with the burial of organic matter in sediments over 3.5 billion years. So this supports the hypothesis that supernovae link to climate, the flux of nutrients, and bioproductivity. It is plausible that there also can be an influence on biodiversity. The present results indeed suggest this. In this manner, astrophysical processes in the form of supernovae appear to have been essential for macroevolutionary changes.

## AUTHOR CONTRIBUTIONS

**Henrik Svensmark:** Conceptualization (lead); data curation (lead); formal analysis (lead); funding acquisition (lead); investigation (lead); methodology (lead); project administration (lead); resources (equal); software (equal); supervision (lead); validation (lead); visualization (lead); writing – original draft (lead).

## CONFLICT OF INTEREST STATEMENT

The author declares no conflict of interest.

## FUNDING INFORMATION

Funding for this project was provided by the Technical University of Denmark.

## Data Availability

Data for this study are stored in Dryad: https://doi.org/10.5061/dryad.2v6wwpzt4
